# Reproductive performance of Norwegian cattle from 1985 to 2005: trends and seasonality

**DOI:** 10.1186/1751-0147-49-5

**Published:** 2007-02-13

**Authors:** Arne Ola Refsdal

**Affiliations:** 1 Geno AI and Breeding, Hamar, Norway

## Abstract

Declining reproductive performance is a serious breeding concern in many countries. To reveal the situation in Norwegian cattle, trends in reproductive performance were studied using insemination reports from 1985 to 2005 and data based on herd recording files from 1989 to 2005. The total number of first services was 469.765 in 1985 declining to 335.712 in 2005. The number of recorded herds and animals declined from 21.588 to 14.718 and 360.289 to 309.452 from 1989 to 2005, respectively. Sixty days non-return rate after single inseminations (NR60) increased from 68.1 in 1985 to 72.7% in 2005 (p < 0.001) and the number of services per inseminated animal (NIA) decreased from 1.8 to 1.6 (p < 0.001) from 1985 to 2005. However, return rates 0–3 days post insemination (RR0-3) increased from 6 to 12% in the same period (p < 0.001). NR60 was higher and the RR0-3 was lower in the summer season compared to the winter season during the whole period. A fertility index (FS), has been calculated from the herd recording files each year from 1989 to 2005. The average FS-index did not show a significant trend and the calving interval was also fairly constant between 12.4 and 12.6 months during this period. The average interval from calving to first and last insemination, respectively, increased from a low of 79 and 102 days in 1990 to a high of 86 and 108 days in 2005. Both intervals were consistently longer for cows in first lactation than for cows in later lactations. The percentage of inseminated animals reported culled because of poor fertility decreased from 6.0% in 1989 to 4.6% in 1996 and thereafter again increased to 6% in 2005. In conclusion, most fertility measures, mainly comprising the Norwegian Red (NRF) breed, show a relatively high level of reproductive performance with a positive or a relatively constant trend during the last two decades.

## Background

In many countries there has been a decline in reproductive performance in dairy cattle. Several studies show increasing number of days from calving to first service and decreasing pregnancy rates, e.g. [1-5]. As a result, the number of inseminations per inseminated cow, days from calving to conception and calving intervals have increased. To improve fertility and save labour, various pharmaceuticals to control the oestrous cycle and to treat reproductive disorders are extensively used in many herds, e.g. [6,7]. During the last decades the productivity of dairy cattle has increased considerably in many countries, not least because of progress due to genetic improvement. However, a serious breeding concern is that estimates from a number of studies present unfavourable genetic correlations, on average near 0.3, between various fertility measures and production [8]. In contrast to many other countries, Norway has a long tradition of including fertility in the Total Merit Index (TMI). Viewed against this background, the primary objective of the present study was to describe the trends in some reproductive measures in Norway the last two decades. Seasonal variations in reproductive performance are also revealed.

## Methods

The results obtained in the present study are based on insemination reports and herd recording files in Norway comprising 66.8% of the herds in 1985 increasing to 94.2% in 2005 [9]. AI-technicians and veterinarians report all inseminations into the AI-database, and they are only paid when the inseminations are registered. From 1985 to 2005, the part of inseminations performed by veterinarians has increased from 45.8% to 59.7%. The rest of the inseminations was performed by technicians, but from 2002 also a small part by herdsmen, increasing from 0.3% to 0.7% in 2005. After attending an AI-course the herdsmen have to sign an agreement to report inseminations to the AI-database. Sixty days non return rates after single inseminations (NR60), return rates 0–3 days post insemination (RR0-3), average number of inseminations per animal inseminated (NIA) and seasonality are based on all inseminations performed in the country during the period, irrespective of membership in the milk recording system. Thus, these data are based on 469.765 number of first services in 1985 [10] declining to 335.712 services in 2005 [11].

Trends concerning age of heifers at first insemination, average number of days from calving to first (CFI) and last insemination (CLI), respectively, number of animals inseminated (I), calving interval and animals culled because of failure to breed (AC) were obtained from herd recording files from 1989 to 2005. During this period there was a decline in number of recorded herds from 21.588 to 14.718 and animals from 360.289 to 309.452. A fertility index, Fertility status (FS), was also calculated for each herd from the herd recording files every year from 1989 to 2005. FS index is expressed by the formula:

(NR60+RR0-3NIA−(125-CLI)) (I-AC)I
 MathType@MTEF@5@5@+=feaafiart1ev1aaatCvAUfKttLearuWrP9MDH5MBPbIqV92AaeXatLxBI9gBaebbnrfifHhDYfgasaacH8akY=wiFfYdH8Gipec8Eeeu0xXdbba9frFj0=OqFfea0dXdd9vqai=hGuQ8kuc9pgc9s8qqaq=dirpe0xb9q8qiLsFr0=vr0=vr0dc8meaabaqaciaacaGaaeqabaqabeGadaaakeaadaWcaaqaamaabmaabaWaaSaaaeaacqqGobGtcqqGsbGucqaI2aGncqaIWaamcqGHRaWkcqqGsbGucqqGsbGucqaIWaamcqqGTaqlcqaIZaWmaeaacqqGobGtcqqGjbqscqqGbbqqaaGaeyOeI0YaaeWaaeaacqaIXaqmcqaIYaGmcqaI1aqncqqGTaqlcqqGdbWqcqqGmbatcqqGjbqsaiaawIcacaGLPaaaaiaawIcacaGLPaaacqqGGaaidaqadaqaaiabbMeajjabb2caTiabbgeabjabboeadbGaayjkaiaawMcaaaqaaiabbMeajbaaaaa@4CBA@

Comparisons of NR60, RR0-3 and NIA between years or groups were performed using chi-square analysis.

## Results

**Figure 1:**
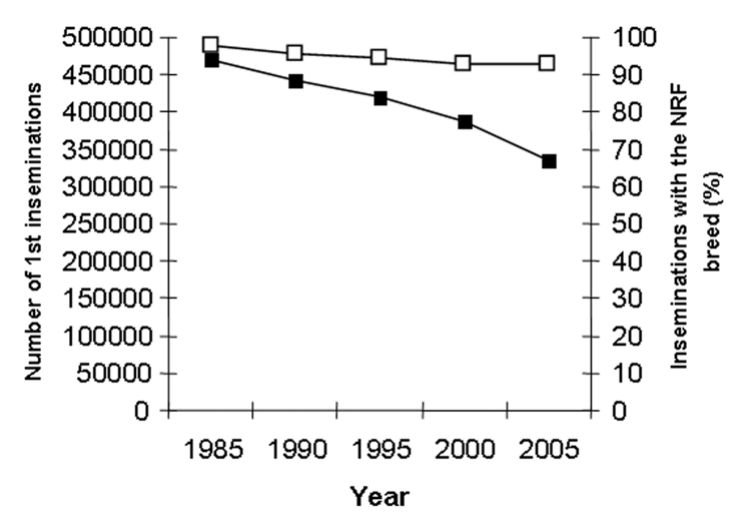
Total number of first inseminations (■) and percentages of inseminations performed with semen from bulls of the NRF breed (□) every 5^th ^year from 1985 to 2005.

**Figure 2:**
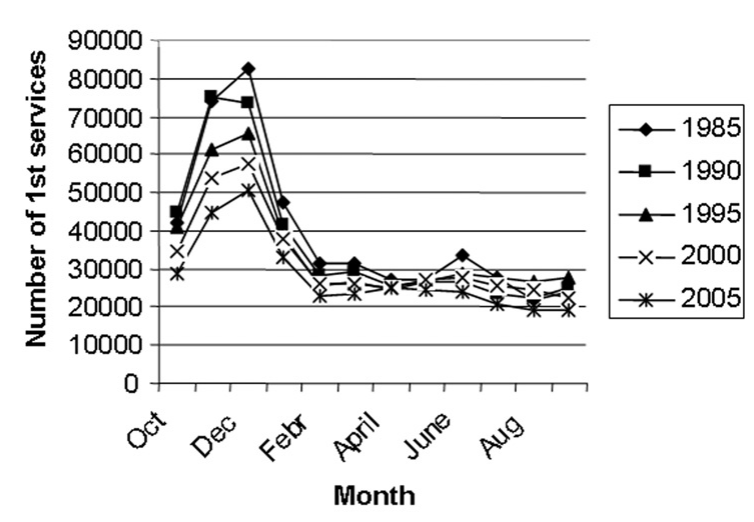
Seasonal distribution of first inseminations every 5^th ^year from 1985 to 2005.

**Figure 3:**
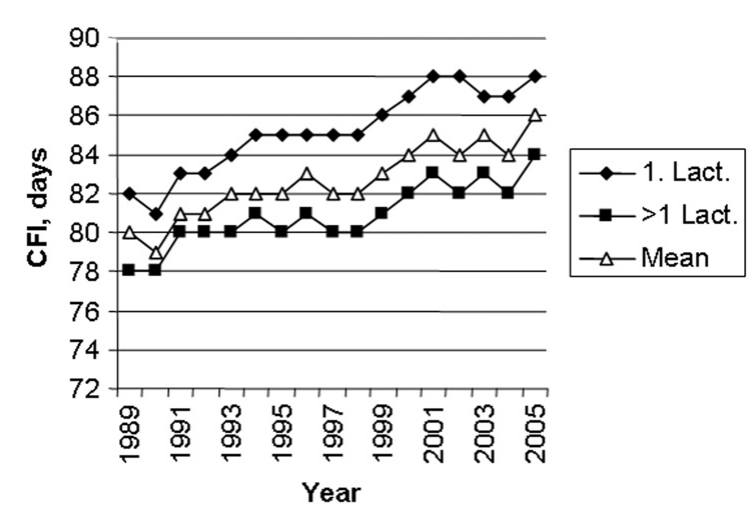
Average interval from calving to first insemination (CFI) in first lactation (◆), later lactations (■) and for all cows (△).

**Figure 4:**
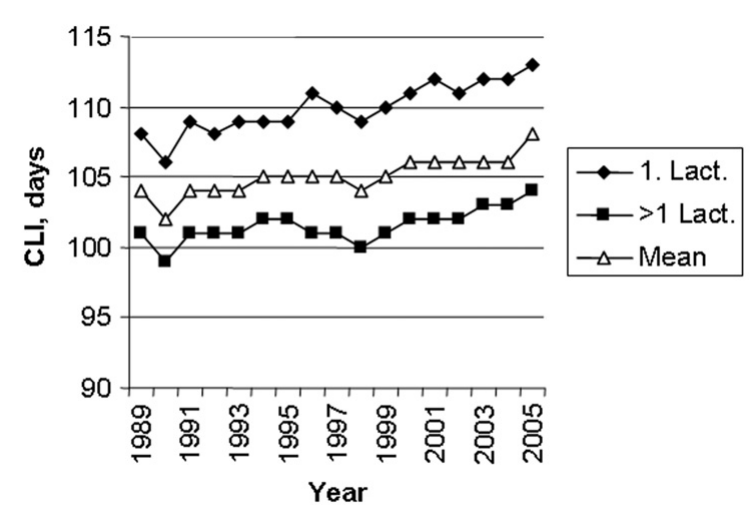
Average interval from calving to last insemination (CLI) in first lactation (◆), later lactations (■) and for all cows (△).

The number of first inseminations every 5^th ^year from 1985 to 2005 is shown in figure 1. The major part of the inseminations is performed with semen from the Norwegian Red (NRF) breed, varying from a high of 97.8% in 1985 to a low of 92.3% in 2003. Semen from other breeds are various beef breeds (4.2% in 2005), mainly used on NRF cattle, and other dairy breeds (3.0% in 2005) [11]. During the period of study, October – January represented the main breeding season with peaks in November and December. From February to September the monthly number of 1^st ^services remained similar (Fig 2). The age of heifers at 1^st ^insemination was at a low of 15.6 months in 1991 and increased to 16.2 months from 2001 to 2005. The average CFI interval has increased from a low of 79 days in 1990 to a high of 86 days in 2005 (Fig 3). The CFI interval for cows in first lactation was consistently longer than for cows in later lactations, increasing from 81 to 88 days and 78 to 84 days respectively, from 1990 to 2005. The average CLI interval has also increased during the period from a low of 102 days in 1990 to a high of 108 days in 2005 (Fig 4). The CLI interval for first lactation cows was also consistently longer than for cows in the second and later lactations, increasing from 106 to 113 days and 99 to 104 days respectively.

**Figure 5:**
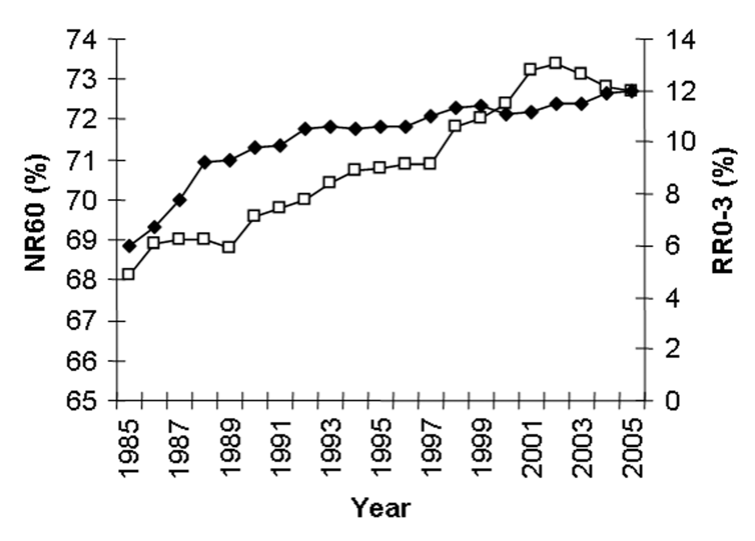
Sixty days non return rates (NR60, □) and return rates within 3 days (RR0-3, ◆) from 1985 to 2005.

**Figure 6:**
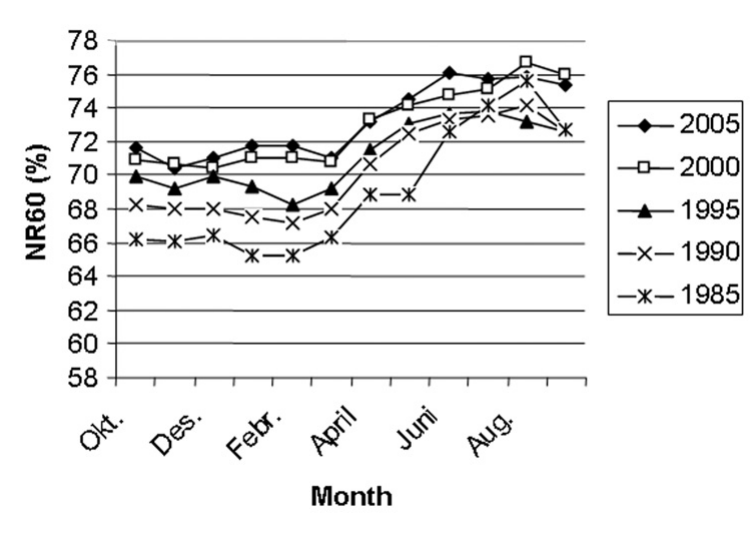
Sixty days non return rates by month every 5th year from 1985 to 2005.

**Figure 7:**
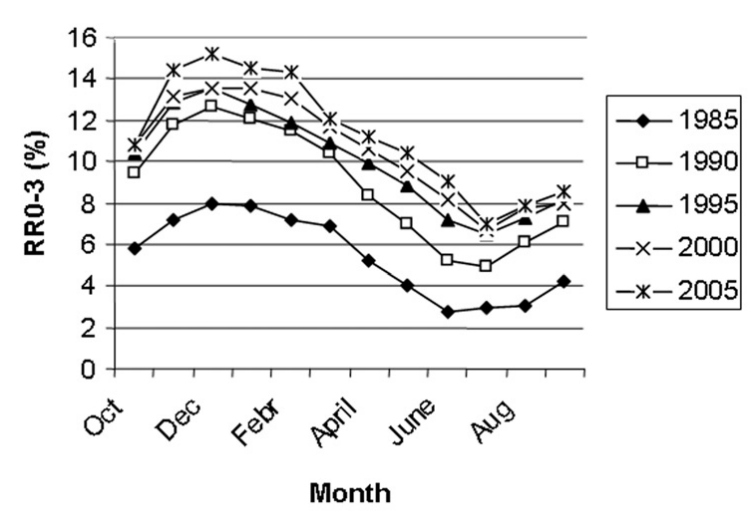
Return rates within 3 days (RR0-3) by month every 5^th ^year from 1985 to 2005.

**Figure 8:**
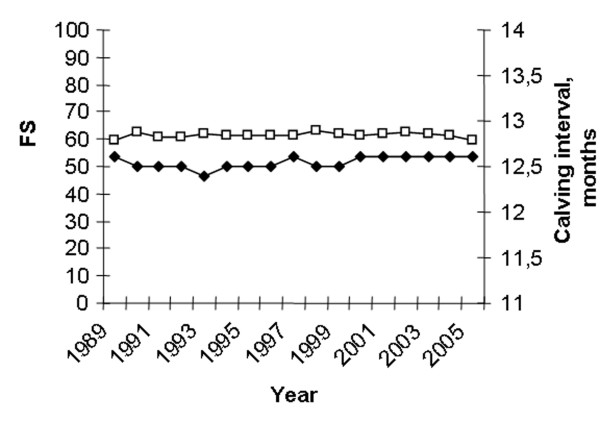
Average FS-index (□) and calving interval (◆) from 1989 to 2005.

Figure 5 shows the trends concerning NR60 and RR0-3. The average NR60 has increased significantly from 68.1% in 1985 to 73.4% in 2002 (p < 0.001) and then declined to 72.7% in 2005. RR0-3 has increased from 6% in 1985 to 12% in 2005 (p < 0.001). The seasonal variation in NR60 every 5^th ^year from 1985 to 2005 is shown in Fig 6. NR60 is consistently higher in the summer than in the winter. However, the difference between the highest summer month and the lowest winter month has decreased substantially from 10.4% in 1985 to 5.7% in 2005. Fig 7 shows the seasonal variation in RR0-3 every 5 year from 1985 to year 2005. RR0-3 is consistently higher during the winter months as compared to the summer months. In 2005 RR0-3 reached a high of 15.2% in December and declined to a low of 7% in July. The overall average NIA has declined from 1.8 in 1985 to 1.6 in 2005. NIA for heifers (n = 96849), cows in 1^st ^lactation (n = 85351) and cows with >1 lactation (n = 127252) were 1.5, 1.8 and 1.7 (P < 0,001) respectively for controlled animals in 2005. Data from 1989 to 2005 show each year similar differences in NIA between heifers, cows in 1^st ^and >1 lactation. Fig 8 shows the average FS-index and calving interval for controlled animals from 1989 to 2005. The FS-index varying between 59.3 (1989) and 63.3 (1998), does not show a specific trend. Average calving interval in controlled animals has varied between 12.4 and 12.6 months during the same period also without showing a specific trend.

**Figure 9:**
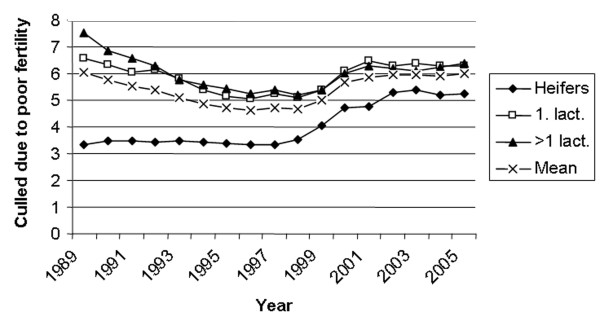
Percentages of inseminated animals reported culled because of poor fertility from 1989 to 2005.

The percentage of inseminated animals reported culled because of poor fertility is shown in Fig 9. The percentage decreased from 6.0% in 1989 to 4.6% in 1996 and thereafter again increased to 6% in 2005. Heifers show lower percentages and had a somewhat different trend compared to lactating animals as they were consistently on about 3.5% from 1989 to 1998 and then increased to a high of 5.4% in 2003.

## Discussion

The aim of the present study was to describe some trends in reproductive measures in Norwegian cattle the last two decades. Since NRF has been by far the most dominant breed during this period, the data presented mainly reflects the reproductive performance of this breed. To describe the fertility trends, 60 days non return rates and number of services per inseminated animal are used among others. As a measure of fertility, non return rates have some disadvantages, as described by Salisbury *et al*. [12]. Cows, once inseminated, may be culled, dead or bred naturally without recording, either on purpose or by accident, and appear in the records as non returns to the original insemination. On the other hand, cows that come in heat and are inseminated while pregnant will appear on the record as returns. This will also be misleading. Moreover, embryonic deaths or abortions cause some cows to return to later service even though they had conceived at an earlier one. However, when applied to large numbers of services like in this study, non return rates are considered to be very useful for studying fertility trends. The registration system in Norway is also considered to be very reliable as the inseminations are performed by technicians employed in one company, Geno (Norwegian breeding and AI-association) and by veterinarians, and both groups are paid by Geno when the inseminations are registered. Reports of inseminations being performed by herdsmen may be somewhat incomplete even though it should be done routinely according to an agreement. However, since inseminations were not performed by herdsmen before 2002 and represent a very small part of the inseminations since then, incomplete reports from this group would be of little significance for the study. Substantially, there have been no changes in the AI reporting routines during the last decades. Therefore, the positive trend in non return rate probably reflects a true fertility improvement. This trend is in accordance with Andersen-Ranberg *et al*., studying phenotypic and genotypic trends in heifers and first lactation cows [13]. However, it is in contrast to a worldwide trend showing a decline in non return rates and pregnancy rates during the last decades, e.g. [2,5]. The non return rates in Norwegian cattle during the last decades probably also reflects a positive trend concerning pregnancy rates. Unfortunately, reliable data to confirm a close trend relationship between the two parameters have not been available so far. However, recently a Norwegian field study has indicated that the pregnancy rate is on average about 12 % lower than overall NR60 after single inseminations [14]. In this study the overall pregnancy rate after single first inseminations in NRF was 60.7%, and the results for heifers, 1^st ^lactation and >1 lactation cows were 68.8, 56.0 and 58.7% respectively. These results show that the pregnancy rates in NRF is relatively high when compared to studies from many other countries, e.g. [15-17].

The improvement in NR60 is probably caused by a variety of reasons, one of them being the breeding strategy, which gives increasing weight to fertility and health traits. In Norway, fertility has been emphasised in the total merit index from the 1970's based on progeny testing utilising large daughter groups of the NRF breed [18]. Other reasons might be different campaigns and courses concerning herd management, nutrition and reproduction, routinely offered to farmers by veterinarians and agricultural advisors. The incidence rate of ketosis has decreased substantially from the mid of the 1980's [19] and this may be an effect of these activities. Reduction of ketosis may have influenced NR60 in a positive way since lowered non return rates have been found in cows treated for ketosis [20]. A successful eradication programme for Bovine Virus Diarrhoea Virus (BVDV) infection that started in Norway in December 1992 could possibly also explain some of the increase in NR60 after that time. BVDV infection is a notifiable disease in Norway, and from the start of the programme the number of restricted herds decreased from a high of 2,950 (11.3% of the herds) in 1994 to 1 by the end of 2005 [21]. BVDV infection has been associated with late return to service [22] and other reproductive disorders [23]. However, in a Norwegian study indications of a reduced conception risk were not detected [24]. From the present knowledge a possible impact of the disease on the NR60 seem to be rather small. In any case, the fact that a relatively low proportion of the herds had restrictions in the beginning of the eradication programme does not make it likely that eradication of BVDV infection is a major cause of the increase of average NR60 that continued after the start of the eradication programme.

The increasing trend in CFI, and consequently also CLI, is probably mainly caused by managerial factors and farmer decisions. However, partly it is probably also caused by a small and undesirable genetic change for CFI, which has been observed in first lactation cows [13]. In this study, the genetic correlation between protein yield and CFI in first lactation was strongly unfavourable. There has been a considerable positive genetic change in protein yield in Norwegian dairy cattle [13]. However, the average milk yield per cow year in the period has increased from 5716 kg to 6541 kg only [9]. Genetically, the breed has a much higher milk yield potential and the relatively low yield is mainly caused by the political framework established in Norway during the period, affecting price mechanisms and feeding regimes. Thus, the system has not favoured high yields. Consequently, use of concentrates during peak lactation may have been limited leading to negative energy balance and longer interval from calving to resumption of regular cyclic ovarian activity and an increasing CFI. CFI and CLI in first lactation animals are longer than for older cows during the observation period. This is probably mainly caused by the fact that many high yielding first lactation cows are less able to meet the nutritional requirements during peak lactation and consequently need more time to resume the ovarian cyclic activity post partum and to show oestrus. However, compared to other studies, the CFI and CLI intervals are relatively short for cows in first and later lactations and the increase of the two parameters during the period studied is relatively moderate, e.g. [4,16]. Use of double inseminations is mainly caused by problems to find the optimum time of insemination. Farmers may realise that they have inseminated animals too early in oestrus and therefore order a second insemination a day or two later. Especially farmers having strictly seasonal calving are dependent on their cows conceiving quickly and therefore may use double insemination in order to be more close to the optimum time in oestrus. The use of double insemination is more pronounced during the winter period than during summer. This may be caused by different environmental conditions, like nutritional management, photointensity and photoperiod during the winter season [25]. Using hormonal treatment to induce or to synchronise oestrus is often followed by a double insemination. This will affect the RR03 and may have caused some of the relatively rapid increase in the use of double inseminations from 1985 to 1990, just after the introduction of prostaglandins in Norway. According to the Norwegian Health Card Statistics, based on records on all milk recorded cows having their own disease journal kept in the barn [26], it was an increase in treatments of cows not observed in heat from 1980 to 1990 [19]. However, since 1990 there has been a decline in the number of such treatments without a concurrent decrease in the use of double inseminations.

The study shows that reproductive performance in Norway is consequently higher in the summer months compared to the winter season. This is in contrast to many countries under subtropical and tropical conditions experiencing decreased fertility in dairy cows inseminated during the hot summer months [27-29]. The opposite situation in Norway is probably caused by a variety of environmental factors, including climatic conditions, light intensity, nutrition (grazing versus indoor feeding) and cattle housing which is different in the relatively cold, temperate climate. Thus, summer heat stress does not seem to cause fertility problems in Norway, but cold and dark winter periods may suppress ovarian activity and oestrus expression and possibly increase embryonic mortality. However, the difference in NR60 between the highest summer months and the lowest winter months has decreased from approximately 10% in 1985 to 5–6% in 2005 as shown in Fig 6. Increasing reproductive performance during winter over the years may be caused by a variety of factors like improved nutritional management during the indoor season and focus on exposing cattle at high latitudes during winter to dim illumination and a minimum photoperiod of 12 h [25]. Another factor may be the female fertility trait, non return rate, being selected for in NRF since 1972. This has resulted in a genetic improvement [18] and probably not least has favoured animals with a high reproductive performance during winter time. The average number of services per animal inseminated has decreased in spite of increasing use of double inseminations during the observation period. The lower number of services in heifers especially compared with 1^st ^lactation animals, but also >1 lactation cows, is in accordance with the differences in pregnancy rates after single first inseminations registered by Refsdal *et al*. [14]. The FS-index has been fairly constant during the observation period even though the NR60 above all has increased. This is mainly caused by the fact that the average CLI interval, which has a great impact in the FS-formula, has increased. Thus, the FS-index takes into account not only the NR60 as a measure of success of insemination, but also the CLI interval reflecting the number of days open which is an economically important factor in milk production. The calving interval has also been fairly constant during the period in spite of increasing NR60 rates. This is also mainly caused by the increase in CLI interval. The percentages of inseminated animals reported culled because of poor fertility are based on information given by the farmers. This information may be inaccurate as farmers may have a different understanding of what is poor fertility, and if there is a combination of different reasons why cows are culled the primary one may be reported more or less by chance. The decline in percentage of animals culled because of poor fertility from 1989 to 1998 is in accordance with the increasing non return rates during the same period while the ensuing increase in culling rate do not correspondingly agree with the non return rates. Per cent of culled cows discarded because of poor fertility was 12.3% in Norway in 2005 [9]. Compared to other studies this is a relatively low percentage [17,30].

## Conclusion

In conclusion, most fertility measures in Norwegian cattle, mainly comprising the NRF breed, show a relatively high level of reproductive performance and a positive (NR60, NIA) or relatively constant trend (Calving interval, FS-index) during the last two decades. This is probably caused by a variety of reasons, one of them being the breeding strategy, which gives increasing weight to fertility and health traits. However, the interval from calving to first and last insemination, respectively, has slightly increased during the period and the RR0-3 has increased. The calving interval has been relatively constant in spite of increasing non return rates and lower number of services per animal inseminated, mainly because of a longer interval from calving to first insemination. This is also the main reason why the FS-index has been relatively constant. In contrast to many countries under subtropical and tropical conditions the reproductive performance in Norway is higher in the summer months compared to the winter season. This pattern has been similar over time and is probably caused by a variety of environmental factors.

## References

[B1] O'Farrell KJ (1998). Changes in dairy cow fertility. Cattle Practice.

[B2] Lucy MC (2001). Reproductive loss in high-producing dairy cattle: where will it end?. J Dairy Sci.

[B3] Washburn SP, Silvia WJ, Brown CH, McDaniel BT, McAllister AJ (2002). Trends in reproductive performance in Southeastern Holstein and Jersey DHI herds. J Dairy Sci.

[B4] Rajala-Schultz PJ, Frazer GS (2003). Reproductive performance in Ohio dairy herds in the 1990s. Anim Reprod Sci.

[B5] McDougall S (2006). Reproduction performance and management of dairy cattle. J Reprod Dev.

[B6] Macmillan KL, Burke CR (1996). Effects of oestrous cycle control on reproductive efficiency. Anim Reprod Sci.

[B7] Roche JF, Austin E, Ryan M, O'Rourke M, Mihm M, Diskin M (1998). Hormonal regulation of the oestrous cycle of cattle. Reprod Domest Anim.

[B8] Philipsson J, Banos G, Arnason T (1994). Present and future uses of selection index methodology in dairy cattle. J Dairy Sci.

[B9] TINE (2006). TINE Årsrapport 2005.

[B10] Geno (1986). Geno Annual Report 1985.

[B11] Geno (2006). Geno Annual Report 2005.

[B12] Salisbury GW, Lodge JR, Van De Mark NL (1978). Physiology of reproduction and artificial insemination of cattle.

[B13] Andersen-Ranberg IM, Klemetsdal G, Heringstad B, Steine T (2005). Heritabilities, genetic correlations, and genetic change for female fertility and protein yield in Norwegian Dairy Cattle. J Dairy Sci.

[B14] Refsdal AO, Karlberg K, Garmo RT (2006). Reproductive performance in the Norwegian Red breed. Reprod Domest Anim.

[B15] Butler WR (1998). Review: effect of protein nutrition on ovarian and uterine physiology in dairy cattle. J Dairy Sci.

[B16] de Vries A, Risco CA (2005). Trends and seasonality of reproductive performance in Florida and Georgia dairy herds from 1976 to 2002. J Dairy Sci.

[B17] Mayne CS, McCoy MA, Lennox SD, Mackey DR, Verner M, Catney DC, McCaughey WJ, Wylie AR, Kennedy BW, Gordon FJ (2002). Fertility of dairy cows in Northern Ireland. Vet Rec.

[B18] Andersen-Ranberg IM, Heringstad B, Klemetsdal G, Svendsen M, Steine T (2003). Heifer Fertility in Norwegian Dairy Cattle: Variance Components and Genetic Change. J Dairy Sci.

[B19] Norwegian Cattle Health Services (2006). Annual Report 2005.

[B20] Refsdal AO, Westerling B (1978). Fertilitetsforhold hos ketosekyr bedømt ut fra helsekortregistreringene i Norge [Fertility in cows treated for ketosis as judged from health card recordings in Norway] English summary. Proceedings of the 13th Nordic Veterinary Congress, 19-22 July 1978, Turku.

[B21] National Veterinary Institute (2006). Surveillance and control programmes for terrestrial and aquatic animals in Norway Annual Report 2005.

[B22] Robert A, Beaudeau F, Seegers H, Joly A, Philipot JM (2004). Large scale assessment of the effect associated with bovine viral diarrhoea virus infection on fertility of dairy cows in 6149 dairy herds in Brittany (Western France). Theriogenology.

[B23] Baker JC (1995). The clinical manifestations of bovine viral diarrhea infection. Vet Clin North Am Food Anim Pract.

[B24] Valle PS, Martin SW, Skjerve E (2001). Time to first calving and calving interval in bovine virus diarrhoea virus (BVDV) sero-converted dairy herds in Norway. Prev Vet Med.

[B25] Reksen O, Tverdal A, Landsverk K, Kommisrud E, Boe KE, Ropstad E (1999). Effects of photointensity and photoperiod on milk yield and reproductive performance of Norwegian red cattle. J Dairy Sci.

[B26] Refsdal AO (2000). To treat or not to treat: a proper use of hormones and antibiotics. Anim Reprod Sci.

[B27] Drost M, Thatcher WW (1987). Heat stress in dairy cows. Its effect on reproduction. Vet Clin North Am Food Anim Pract.

[B28] Wolfenson D, Roth Z, Meidan R (2000). Impaired reproduction in heat-stressed cattle: basic and applied aspects. Anim Reprod Sci.

[B29] Rensis FD, Scaramuzzi RJ (2003). Heat stress and seasonal effects on reproduction in the dairy cow--a review. Theriogenology.

[B30] Esslemont RJ, Kossaibati MA (1997). Culling in 50 dairy herds in England. Vet Rec.

